# International Guidelines for the Diagnosis and Management of Hyperinsulinism

**DOI:** 10.1159/000531766

**Published:** 2023-07-14

**Authors:** Diva D. De Leon, Jean Baptiste Arnoux, Indraneel Banerjee, Ignacio Bergada, Tricia Bhatti, Louise S. Conwell, Junfen Fu, Sarah E. Flanagan, David Gillis, Thomas Meissner, Klaus Mohnike, Tai L.S. Pasquini, Pratik Shah, Charles A. Stanley, Adrian Vella, Tohru Yorifuji, Paul S. Thornton

**Affiliations:** a Congenital Hyperinsulinism Center and Division of Endocrinology and Diabetes, Department of Pediatrics, Children’s Hospital of Philadelphia, Perelman School of Medicine at the University of Pennsylvania, Philadelphia, PA, USA;; b Reference Center for Inherited Metabolic Diseases, Necker-Enfants Malades Hospital, AP-HP, University of Paris-Cité, Paris, France;; c Paediatric Endocrinology, Royal Manchester Children’s Hospital, University of Manchester, Manchester, UK;; d Centro de Investigaciones Endocrinológicas “Dr. César Bergadá” (CONICET – FEI), Division de Endrocrinología, Hospital de Niños Ricardo Gutiérrez, Buenos Aires, Argentina;; e Department of Clinical Pathology and Laboratory Medicine, Children’s Hospital of Philadelphia and Perelman School of Medicine at the University of Pennsylvania, Philadelphia, PA, USA;; f Australia and Children’s Health Queensland Clinical Unit, Department of Endocrinology and Diabetes, Queensland Children’s Hospital, Children’s Health Queensland, Greater Brisbane Clinical School, Medical School, Faculty of Medicine, University of Queensland, Brisbane, QLD, Australia;; g National Clinical Research Center for Child Health, Department of Endocrinology, The Children’s Hospital of Zhejiang University School of Medicine, Hangzhou, China;; h Institute of Biomedical and Clinical Science, University of Exeter Medical School, Exeter, UK;; i Hadassah Medical Center, Department of Pediatrics, Ein-Kerem, Jerusalem and Faculty of Medicine, Hebrew-University, Jerusalem, Israel;; j Department of General Pediatrics, Neonatology and Pediatric Cardiology, University Children’s Hospital, Medical Faculty, Heinrich Heine University, Duesseldorf, Germany;; k Department of General Pediatrics, Otto-von-Guericke University Magdeburg, Magdeburg, Germany;; l Research and Policy Director, Congenital Hyperinsulinism International, Glen Ridge, NJ, USA;; m Pediatric Endocrinology, The Royal London Children’s Hospital, Queen Mary University of London, London, UK;; n Division of Diabetes, Endocrinology and Metabolism, Mayo Clinic, Rochester, MN, USA;; o Pediatric Endocrinology and Metabolism, Children’s Medical Center, Osaka City General Hospital, Osaka, Japan;; p Congenital Hyperinsulinism Center, Cook Children’s Medical Center and Texas Christian University Burnett School of Medicine, Fort Worth, TX, USA

**Keywords:** Hyperinsulinism, Guidelines, Hypoglycemia, Insulin

## Abstract

**Background:**

Hyperinsulinism (HI) due to dysregulation of pancreatic beta-cell insulin secretion is the most common and most severe cause of persistent hypoglycemia in infants and children. In the 65 years since HI in children was first described, there has been a dramatic advancement in the diagnostic tools available, including new genetic techniques and novel radiologic imaging for focal HI; however, there have been almost no new therapeutic modalities since the development of diazoxide.

**Summary:**

Recent advances in neonatal research and genetics have improved our understanding of the pathophysiology of both transient and persistent forms of neonatal hyperinsulinism. Rapid turnaround of genetic test results combined with advanced radiologic imaging can permit identification and localization of surgically-curable focal lesions in a large proportion of children with congenital forms of HI, but are only available in certain centers in “developed” countries. Diazoxide, the only drug currently approved for treating HI, was recently designated as an “essential medicine” by the World Health Organization but has been approved in only 16% of Latin American countries and remains unavailable in many underdeveloped areas of the world. Novel treatments for HI are emerging, but they await completion of safety and efficacy trials before being considered for clinical use.

**Key Messages:**

This international consensus statement on diagnosis and management of HI was developed in order to assist specialists, general pediatricians, and neonatologists in early recognition and treatment of HI with the ultimate aim of reducing the prevalence of brain injury caused by hypoglycemia. A previous statement on diagnosis and management of HI in Japan was published in 2017. The current document provides an updated guideline for management of infants and children with HI and includes potential accommodations for less-developed regions of the world where resources may be limited.

## Method of Development of Evidence-Based Clinical Practice Guidelines

The guideline-writing committee comprised a group of 17 members including 13 pediatric endocrinologists, an adult endocrinologist, a pathologist, a genetic scientist, and a representative of an international patient advocacy organization for HI. Following a preparatory meeting (September 2019), working groups were assigned, and each undertook a literature review. The combined work was collated into a single document which was revised by all members of the writing committee over a 3-year period. Participating pediatric endocrine societies of the International Consortium of Pediatric Endocrinology (ICPE) were invited to review the document with feedback incorporated into the final version. The evidence was graded using the framework of Grading of Recommendations, Assessment, Development, and Evaluation (GRADE), describing both the strength of recommendations and the quality of the evidence [1]. A detailed description of the grading scheme has been previously published [2].

In terms of strength of recommendations, strong recommendations used the phrase “‘we recommend’” and the number 1, and conditional recommendations used the phrase “‘we suggest’” and the number 2. Cross-filled circles indicate the quality of the evidence such that ⊕○○○ denotes very low-quality evidence; ⊕⊕○○, low-quality; ⊕⊕⊕○, moderate-quality, and ⊕⊕⊕⊕, high-quality. In the absence of sufficient evidence, conclusions were based on expert opinion.

High-quality evidence is defined as “well-performed Randomized Controlled Trials (RCTs) or very strong evidence from unbiased observational studies”; moderate-quality is defined as “RCTs with some limitations or strong evidence from unbiased observational studies”; low-quality is defined as “RCTs with serious flaws or some evidence from observational studies”; and very low-quality is defined as “unsystematic clinical observations or very indirect evidence observational studies” [2].

## 1. The Diagnosis of Hyperinsulinism

### 1.1 We Recommend Making a Specific Diagnosis of Hyperinsulinism Based on Measurements, at a Time of Hypoglycemia, of Plasma Levels of Metabolic Fuels (Beta-Hydroxybutyrate [BOHB] and Free Fatty Acids [FFA]) and Hormones (Insulin, Growth Hormone, Cortisol) and Determination of the Glycemic Response to a Pharmacologic Dose of Glucagon [Grade 1 ⊕⊕⊕○]

The diagnosis of HI is made on the basis of increased insulin action and/or inadequate suppression of plasma insulin during either spontaneous or fasting-induced hypoglycemia. Insulin should be measured using a high-sensitivity assay [3]. Increased insulin action can be demonstrated by increased glucose requirement (e.g., >8 mg/kg/min in a neonate normal 4–6 mg/kg/mi [4]), inappropriately suppressed plasma concentrations of FFA and BOHB during hypoglycemia, and an inappropriate glycemic response to glucagon at a time of hypoglycemia (Table 1) [5–7]. Suppression of BOHB and preservation of a large glycemic response to glucagon are particularly sensitive markers of inappropriate insulin action. In the absence of multiple pituitary hormone deficiencies in neonates, an inappropriately large glycemic response to glucagon stimulation may be considered diagnostic, particularly when plasma insulin concentration is low (Table 1). Newborn infants, tested before 72 h of life during the time of transitional hypoglycemia, should be retested after 72 h if hypoglycemia is still present to confirm the diagnosis [8]. Provocative stimulation tests with glucose, leucine, or protein are not useful for establishing the diagnosis of HI but may be helpful in defining the subtype of HI [9–11]. Occasionally, there will be a discrepancy between markers of insulin action and insulin levels with elevated insulin levels in the presence of high ketones and absent glucagon response. In these cases, it is possible the insulin assay is not as sensitive as it could be, and the focus should be on the markers of insulin action, not the insulin level itself.

1.2 We Recommend Genetic Testing for all Children except for Those Likely to Have an Acquired Form of Hyperinsulinism [GRADE 1⊕⊕⊕○]

1.3 We Recommend Evaluation of Infants with Hyperinsulinism for Multi-System Syndromes Associated with Hyperinsulinism [GRADE 1⊕⊕○○]

### 1.4 We Recommend Screening all Children Whose Hyperinsulinism Occurs after the Age of 2 years of Life for Insulinoma [Grade 1⊕⊕○○]

It is important to determine the precise etiology of HI because the diagnosis may direct choice of therapy and need for long-term follow-up [12]. HI presenting in the immediate neonatal period may be caused by either genetic or acquired conditions (Table 2). Acquired HI may be secondary to perinatal factors such as maternal diabetes, perinatal stress, birth asphyxia, intrauterine growth restriction, exposure to maternal drugs, or high rates of maternal glucose infusions during delivery. Cases of Perinatal Stress-Induced Hyperinsulinism (PSHI) typically present in the first 24 h of life and affect approximately 1 in 1,200–1,700 newborns [13, 14]; they often resolve within the first 10–14 days of life. PSHI is an exaggerated form of transitional hypoglycemia triggered by hypoxia-mediated reduction of the beta-cell glucose threshold for suppression of insulin secretion [8, 15, 16]. In approximately 1 in 12,000–13,600 newborns, a more severe form of PSHI occurs which persists beyond the first 2 weeks of life and may require treatment with diazoxide [17, 18]. Genetic testing is not usually recommended for infants with PSHI because there is no current evidence of a genetic etiology.

When there is a family history suggestive of maturity-onset diabetes of the young (MODY), genetic testing for *HNF1A* and *HNF4A* should be considered because these conditions can cause transient hyperinsulinism in the newborn period [19–21]. In children older than 2 years of age with acquired HI, the possibility of an insulinoma should be considered [22, 23]. Insulinomas are usually solitary and benign, but they can be multiple and are rarely malignant. Insulinomas can be a component of multiple endocrine neoplasia type 1, and genetic investigations should be directed accordingly [24].

**Fig. 1.**
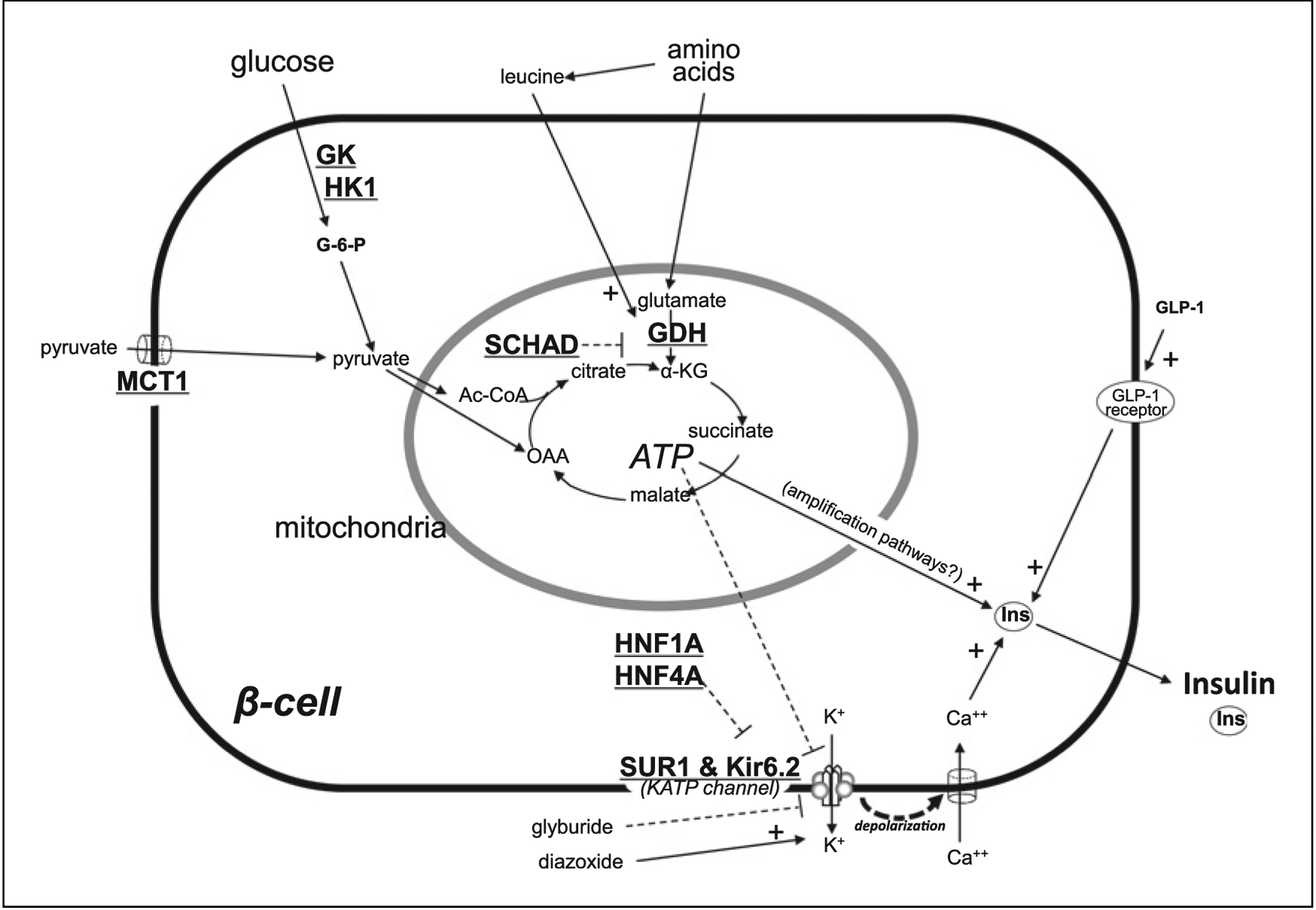
Schematic beta-cell with pathways involved in insulin secretion. Pathways of glucose and amino acid stimulation of beta-cell insulin secretion showing sites of the more common genetic forms of hyperinsulinism, such as the ATP-sensitive (K_ATP_) potassium channel, comprised of SUR1 and Kir6.2 subunits encoded by the *ABCC8* and *KCNJ11* genes. Most genetic forms of hyperinsulinism affect steps by which increased ATP generation closes K_ATP_ channels to depolarize the plasma membrane and activate an influx of calcium to “trigger” release of insulin from storage granules into the circulation. Additional factors, such as stimulation by the gut incretin, GLP1, can act downstream of the triggering pathway to “amplify” insulin release. Drugs such as diazoxide and glyburide bind to the KATP channel to inhibit or activate insulin release, respectively. GK, glucokinase; HK1, hexokinase 1; GDH, glutamate dehydrogenase; SCHAD, short-chain acyl-CoA dehydrogenase; MCT1, mono-carboxylate transporter 1; ATP, adenosine triphosphate; HNF1A & HNF4A, hepatic nuclear factors 1 alpha and 4 alpha; SUR1, sulfonylurea receptor 1; Kir6.2, potassium channel subunit; K_ATP_ channel, ATP-sensitive potassium channel; K^+^, potassium ion; Ca^++^, calcium ion; Ins, insulin; GLP1, glucagon-like peptide 1. Dotted line with dash indicates inhibit, Solid line with + indicates stimulates.

Genetically inherited forms of HI cause isolated pancreatic dysfunction but also may be associated with syndromes affecting multiple organs (Syndromic HI) (Table 3). Genetic forms of HI may present in the newborn period, but may not be detected until later in life. Non-syndromic genetic HI is estimated to occur in approximately 1:25,000–1:45,000 newborns [70–73]. Loss-of-function variants in the *ABCC8* and *KCNJ11* genes, which encode the beta-cell ATP-sensitive potassium (K_ATP_) channel (shown in Fig. 1), are the most frequent cause [74]. Most recently, heterozygous non-coding variants which prevent silencing of Hexokinase 1 within the pancreatic beta-cell have also been shown to be an important cause of isolated HI [75]. Certain genetic types of HI have characteristic phenotypes that can assist in their diagnosis; glutamate dehydrogenase (GDH) HI is associated with moderately elevated plasma ammonia concentrations [76] and short-chain hydroxyacyl-CoA dehydrogenase (SCHAD) HI may be associated with elevated plasma C4-OH acyl carnitine and urine 3-OH-glutarate levels [77]. Protein-induced hypoglycemia is part of the phenotype in GDH-HI, SCHAD-HI, and K_ATP_-HI [11, 60, 78]. Anaerobic exercise can induce hypoglycemia in some patients with activating variants in the promoter region of *SLC16A1*, the gene encoding the monocarboxylate transporter 1 (MCT1) pyruvate transporter reported in a small number of cases [79]. Patients with glucokinase (GCK) HI may develop ketotic hypoglycemia with prolonged fasting, in contrast to the hypoketotic hypoglycemia usually found in HI, especially in the setting of glucose levels dropping below the altered threshold for insulin secretion [27]. Patients with somatic mutations associated with localized islet nuclear enlargement (LINE) pathology usually present at a later age [80].

#### Alternate Classification of HI

HI may usefully be classified according to the response to diazoxide, the first-line drug for controlling hypoglycemia in children with HI. In the diazoxide-unresponsive group up to 90% of cases have pathogenic variant(s) in *ABCC8 or KCNJ11* [74] with diffuse or focal pancreatic histopathology. Recessive bi-allelic variants or dominant mono-allelic *ABCC8 or KCNJ11* pathogenic variants cause diffuse HI. In contrast, focal HI results from the combination of a paternally inherited recessive *ABCC8 or KCNJ11* pathogenic variant and paternal isodisomy of the 11p15 chromosomal region confined to the pancreatic lesion [81]. In some cases, paternal isodisomy of chromosome 11p15 occurs in multiple tissues, causing Beckwith-Wiedemann Syndrome spectrum (BWSp); severe diazoxide-unresponsive HI can occur in BWSp due to 11pUPD when there is a concurrent paternally inherited *ABCC8* or *KCNJ11* disease-causing variant [82]. Therefore, diazoxide unresponsiveness indicates a higher likelihood of finding a genetic etiology. The classification of HI into diazoxide responsive or not presumes access to and treatment with diazoxide. However, in some circumstances, diazoxide may not be available, or the person with HI may be intolerant or experience unacceptable side effects. In such cases, the decision to undertake genetic testing should be made on the merits of the individual case.

Some “atypical” histological forms of HI with localized islet pancreatic involvement (named Localized Islet Nuclear Enlargement [LINE] HI or “mosaic” HI) are caused by somatic dominant variants of the *ABCC8* and *GCK* genes [80, 83–86]. Increased Hexokinase 1 (*HK1*) protein expression has also been identified in pancreatic tissue, suggesting a role for pancreatic *HK1* in HI pathogenesis [85].

Genetic testing is recommended whenever acquired HI is unlikely. This should include children with diazoxide-unresponsive HI as well as those with diazoxide-responsive HI which persists beyond the first 3 months of life. Rapid testing of the *ABCC8* and *KCNJ11* genes is crucial for the management of children with diazoxide-unresponsive HI because the presence of a paternally inherited pathogenic variant predicts a focal lesion with a sensitivity of 97% [74]. Pancreatic imaging (see Recommendation 1.5 below) can then be performed to localize the lesion prior to surgery which is curative in the majority of cases. For centers unable to perform such imaging, rapid genetic testing of *ABCC8* and *KCNJ11* will provide information on cases likely to benefit from transfer to a center with appropriate imaging and surgical capacities [87]. If access to rapid testing of these genes is limited, effort should be made to contact one of several international laboratories able to screen the *ABCC8* and *KCNJ11* genes, sometimes at reduced cost on compassionate grounds.

When no pathogenic variant in *ABCC8* or *KCNJ11* is found on rapid testing or when HI has persisted beyond 3 months, genetic testing of all known HI genes should be performed. The current method of choice is next-generation sequencing by either targeted panel analysis or whole exome/genome sequencing since these provide rapid and cost-effective screening of multiple genes in parallel. It is important to emphasize that no single test is able to detect all forms of genetic variation reported in HI [88]. For example, Sanger sequencing is unable to detect large copy number variants that may occur in some HI genes; in addition, non-coding variants deep within intronic regions will not be detected by exome sequencing or gene panels that do not target these regions (e.g., deep intronic variants in *ABCC8*, *HADH*, and *HK1*) [89]. Also important to note is that separate methylation testing for imprinting defects in the 11p BWS region will be required for children with clinical suspicion of BWSp [90].

### 1.5 We Recommend that Pancreatic Imaging Studies (e.g., ^18^F-DOPA PET Scan) to Localize a Potentially Resectable Focal Lesion Be Performed in Infants with Diazoxide-Unresponsive Hyperinsulinism, except in Those with Genetic Evidence of Diffuse Disease [GRADE 1 ⊕⊕⊕○]

The finding of a single paternally-inherited recessive *ABCC8* or *KCNJ11* pathogenic variant offers a positive predictive value up to 94% for focal HI [74, 91]. For these infants and for diazoxide-unresponsive infants with negative or inconclusive genetic testing, we recommend imaging of the pancreas using 6-fluoro-(^18^F)-L-3,4-dihydroxyphenylalanine positron emission tomography (^18^F-DOPA PET scan) in combination with CT or MRI to help localize the focal lesion [92, 93]. Published studies of 286 histologically assessed cases have reported a sensitivity ranging from 75% to 100% and specificity ranging from 88% to 100% for focal HI [93]. When a focal lesion is detected, the accuracy of localization with ^18^F-DOPA PET scan is greater than 90% [93], but it is highly dependent on the experience of the reader. Very small lesions may not be detected by ^18^F-DOPA PET scan; thus, a negative study does not rule out focal HI. For infants with genetic evidence of diffuse disease (e.g., dominant variants, bi-allelic K_ATP_ channel variants, or glucokinase variants), there is no need for pancreatic imaging with an ^18^F-DOPA PET scan. Recently, 68Gallium-NOGADA-exendin-4-PET/CT has been reported as a beta-cell-specific tracer but has to be further evaluated before being recommended as an additional option [94]. Imaging of the pancreas using conventional techniques such as ultrasound, computerized tomography (CT), and magnetic resonance imaging (MRI) is not helpful in detecting focal lesions in children with congenital HI and should be restricted to older children in whom an acquired insulinoma is suspected.

## 2. Medical Management

### 2.1 We Recommend that the Goal of Treatment for Hyperinsulinism Is to Maintain Plasma Glucose Concentrations within the Normal Range of 70–100 mg/dL (3.9–5.6 mmol/L) [Grade 1⊕⊕○○]

The immediate goal of therapy is to promptly restore plasma glucose to the normal range of 70–100 mg/dL (3.9–5.6 mmol/L) [95]. Following stabilization of glucose, efforts should be directed at identifying the optimal treatment regimen according to the type of HI. The Pediatric Endocrine Society (PES) guidelines established a treatment goal of >70 mg/dL (3.9 mmol/L) for the management of children with hypoglycemia disorders in order to prevent hypoglycemia unawareness [95] and to have a safe margin above the threshold of hypoglycemia that causes brain damage.

Despite attempts to meet the PES glycemic targets, studies have shown that glucose levels may be found below this range (70–100 mg/dL, 3.9–5.6 mmol/L) in children with severe forms of hyperinsulinism in up to 15–20% measurements [96]. In circumstances such as these, a lower hypoglycemia threshold of 63 mg/dL (3.5 mmol/L) may need to be accepted in individual patients, depending on the severity and frequency of hypoglycemia and the availability of alternative treatment.

The frequency of monitoring for the presence of hypoglycemia by glucometer testing should be tailored to the individuals’ needs with typical monitoring before meals and bedtime supplemented by additional testing when needed. There is not enough current evidence to make a suggestion regarding the use of continuous glucose monitoring by subcutaneous sensors.

### 2.2 We Recommend that Intravenous Glucose (Dextrose) Infusion Be Used as the Initial Treatment to Promptly Restore Euglycaemia for Neonates with Hyperinsulinism [Grade 1 ⊕⊕⊕○]

Intravenous glucose (dextrose) should be used for initial treatment of hypoglycemia to restore plasma glucose levels to the normal range without delay. The usual dose is 200 mg/kg (2 mL/kg of 10% dextrose solution), followed by continuous infusion of dextrose at a rate sufficient to maintain plasma glucose above 70 mg/dL (typically 8 mg/kg/min or greater) [97]. In cases where the child is known to have hypoglycemia caused by hyperinsulinism and IV therapy is not immediately available, pharmacologic doses of glucagon (0.5–1 mg or 20–30 μg/kg) can be used as an alternative acute therapy, given either intramuscularly or subcutaneously, with an effect lasting up to 40–60 min allowing time for IV insertion [95]. Since the rates of glucose consumption can be as high as 20–30 mg/kg/min in infants with HI, rapid increases in the glucose infusion rate (GIR) may be required. In such children, with clear evidence of increased glucose utilization, we suggest rapidly increasing glucose infusion rates by increments of 4 mg/kg/min or greater, taking care not to induce fluid overload [98]. Following a 200 mg/kg dextrose bolus and an infusion of 8 mg/kg/min dextrose steady state levels in glucose are reached in 10 min, allowing repeat testing to be accurate and avoid over treatment [99]. To avoid fluid overload, central venous catheters may be required in order to provide dextrose solutions in high concentration.

### 2.3 We Recommend Continuous Intravenous Infusion of Glucagon for Infants at Risk of Fluid Overload because of a High Glucose Requirement [Grade 1⊕⊕○○]

When high-dose intravenous glucose is required, continuous intravenous infusion of glucagon as an additional therapy can help control hypoglycemia and prevent complications from fluid overload by reducing the rate of intravenous dextrose infusions required to prevent hypoglycemia [100]. An intravenous infusion of glucagon (dose range 2.5–20 μg/kg/h) can aid in maintaining euglycemia in HI by stimulation of hepatic glucose production from glycogenolysis and inhibition of hepatic glucose uptake [101, 102]. Adverse side effects of glucagon infusion may include vomiting (13%), rash (2%), and respiratory distress (19%) [100]. A rare but important side effect associated with glucagon is a necrolytic migratory erythema skin rash (NME) [103, 104]. Long-term continuous infusion of glucagon by subcutaneous pumps has been reported but is currently unreliable because of the formation of fibrils and crystals that block infusion lines [105].

### 2.4 We Recommend Diazoxide as the First-Line Treatment for Patients with an Established Diagnosis of Hyperinsulinism [Grade 1⊕⊕⊕○]

Diazoxide is a benzothiadiazide related to thiazide diuretics that was initially developed for treatment of hypertension but later found to be useful to treat HI due to its suppressive effect on insulin secretion [106]. In 1976, diazoxide was approved in the USA by the Food and Drug Administration (FDA) for HI in children. Diazoxide suppresses insulin secretion by opening the beta-cell K_ATP_ channels [107]. The therapeutic dose range of diazoxide in infants and children is 5–15 mg/kg/day po with higher doses offering no additional benefits but greater side effects. In adults, a dose range of 3–8 mg/kg/day is recommended (diazoxide prescribing information, accessdata.fda.gov). The half-life of diazoxide in children was recently estimated to be 15 ± 5.3 h [108]; thus, diazoxide may be administered two or three times daily. To avoid diazoxide-associated fluid retention, particularly in newborn infants [109, 110], a diuretic should be started concomitantly with the initiation of diazoxide. Doses of chlorothiazide of 10 mg/kg/day or hydrochlorothiazide of 1–2 mg/kg/day may be used, particularly when giving higher (>10 mg/kg/day) doses of diazoxide [111].

Responsiveness to diazoxide can be demonstrated by showing that the cardinal feature of HI, hypoketotic hypoglycemia, has been reversed. In practice, this means demonstrating that the infant or child can fast and generate hyperketonemia (BOHB levels >1.8 mmol/L) prior to developing hypoglycemia (plasma glucose levels below 50–60 mg/dL [<2.8–3.3 mmol/L]) [112]. In patients in whom the rate of dextrose infusion cannot be reduced after 5 days of treatment with diazoxide at a dose of 15 mg/kg/day or in whom unresponsiveness has been confirmed by a fasting test, diazoxide should be discontinued. Unresponsiveness to diazoxide suggests a K_ATP_ channel defect [74], although other genetic forms of HI may also be diazoxide-unresponsive, such as glucokinase HI and hexokinase HI.

The acute side effects of diazoxide include salt and water retention which may lead to fluid overload, edema, hyponatremia, tachypnea, and respiratory failure [109]. Pulmonary hypertension has also been described with diazoxide [113–115]; in a large cohort of infants [109] the frequency of diazoxide-related pulmonary hypertension was 2.4%. The most common long-term side effect of diazoxide is hypertrichosis recently reported to occur in 84.1% of children [116]. Coarsening of facial features has been reported in 24% [111, 117]. Other rarer adverse events include neutropenia (15.6%), thrombocytopenia (4.7%), and hyperuricemia (5.0%). The clinical significance of the neutropenia is not known, and the level of neutropenia that deserves discontinuation of diazoxide is also not known. A CBC may be drawn prior to starting diazoxide to ensure there is no evidence of pre-existing neutropenia or thrombocytopenia. The overall frequency of serious adverse events requiring diazoxide discontinuation is estimated at 9.7% [110]. The rate of side effects appears to be higher in infants treated for perinatal stress-induced HI or premature babies [110]. For these reasons, screening for side effects during diazoxide therapy is recommended, including an echocardiogram 1 week after initiation of therapy with diazoxide and for symptoms of pulmonary hypertension. In addition, a complete blood count with differential and serum uric acid levels should be done every 6 months [111]. While there are no published data to evaluate the clinical significance of elevated uric acid levels associated with the use of diazoxide, studies in other populations have shown that chronic hyperuricemia in children can lead to monosodium urate deposits that may progress to gout, just as in adults [118]. Thus, we consider it important to screen for hyperuricemia in children treated with diazoxide given the reported 5% frequency of hyperuricemia in this population [109].

### 2.5 We Suggest the Use of Somatostatin Analogues as Second-Line Treatment for Infants with Hyperinsulinism Who Are Diazoxide-Unresponsive or Have Unacceptable Diazoxide Side Effects or Are Unable to Obtain Diazoxide [Grade 2⊕⊕○○]

Short- or long-acting somatostatin analogs (SSA) used in patients with HI include octreotide [119, 120], long-acting octreotide (octreotide LAR) [121, 122], and lanreotide [122–126]. Octreotide has been used since the late 1980s as a long-term therapy for HI to avoid the need for pancreatectomy or in cases that could not be controlled following subtotal pancreatectomy [119, 120, 127, 128, 129]. However, the use of octreotide therapy is limited by loss of efficacy due to tachyphylaxis [120, 130] and the occurrence of important side effects [131–134], including necrotizing enterocolitis (NEC), particularly in premature and high-risk infants with hemodynamic instability or sepsis [132, 135–137]. While somatostatin analogs are commonly used as second-line treatment of HI, this indication has not been approved by the United States FDA.

Octreotide can be administrated in 2–4 subcutaneous doses/day or by continuous subcutaneous infusion using commercial pumps intended for insulin administration [127, 129]. The starting dose of octreotide is 5–10 μg/kg/day, and it can be titrated up to a maximum of 20 μg/kg/day [138, 139]. In an effort to prevent the development of tachyphylaxis, some centers use two doses of octreotide during the daytime in combination with continuous overnight dextrose administered through a gastrostomy tube [35].

A single monthly injection of a long-acting SSA can have a therapeutic advantage over multiple daily octreotide injections or continuous subcutaneous octreotide by a pump with attendant risk of pump disconnection and failure. The dose of long-acting SSA preparations required to control hypoglycemia is variable, and there are insufficient data available to make a recommendation, but expert consensus suggests calculating the total monthly dose of octreotide and administrating it as a single dose of Octreotide LAR once per month. For lanreotide, the calculation is not equivalent; thus, 30–60 mg once per month is a typical starting dose. Screening for side effects while on SSA therapy is recommended, including growth monitoring, obtaining a gall bladder ultrasound to evaluate for cholelithiasis, and laboratory evaluation for liver enzymes, growth factors, and thyroid function at least every 6 months.

### 2.6 We Suggest that Medications Which Lack Adequate Proof of Efficacy (i.e., Nifedipine, Sirolimus, etc.) Not Be Used to Treat Hyperinsulinism unless Part of an Approved Investigational Protocol [Grade 2⊕⊕○○]

Despite in vitro studies showing inhibition of insulin secretion by calcium-channel blockers and early reports suggesting beneficial effects of nifedipine in infants with HI, most major centers have not found it useful and do not recommend its use [140]. However, in patients with HI due to genetic defects of the *CACNA1D* calcium channel, it has been suggested that nifedipine may improve not only hypoglycemia but also neuromuscular manifestations [141, 142].

Sirolimus, an inhibitor of mammalian target of rapamycin (mTOR), has been used in some children with HI resistant to conventional medical therapies, based on reports of beneficial responses in adults with insulinoma [143]. However, due to serious concerns over the risk of life-threatening infections, such as hepatitis, diabetes mellitus, and pancreatic insufficiency [144–148], and the absence of robust clinical effectiveness data, the Guideline Committee cannot recommend sirolimus for routine clinical use in HI. Exceptions should only be made for studies of the drug carried out under approved investigational protocols. Glucocorticoids have been used in the past in an effort to increase glucose levels by inducing insulin resistance; however, we suggest that they are not effective in treatment of HI and should be avoided since the risks of this therapy strongly outweigh any benefits.

### 2.7 We Suggest Carbohydrate Supplementation to Maintain Euglycaemia in Infants and Children with Hyperinsulinism Who Are Not Adequately Controlled on Pharmacologic Therapy Alone [Grade 2⊕⊕○○]

Many children with HI continue to have unstable hypoglycemia requiring continuous intravenous treatment with glucose despite maximal medical and/or surgical treatment. For these children, continuous intragastric infusion of glucose may be used, either via nasogastric tube or, preferably, via gastrostomy using portable pumps [149]. Solutions containing glucose (dextrose or maltodextrin polymer powder) at concentrations up to 20% may be tolerated. In some cases, glucose supplementation of formula feedings to increase the total carbohydrate content to 15% may be helpful [150]; however, care should be taken when using glucose supplementation to avoid interfering with appetite and normal feeding behavior or inducing obesity due to the excess calories [151].

Some reports have suggested that drinks containing uncooked cornstarch may increase fasting tolerance in older children and adults with HI who have unstable control of hypoglycemia, similar to the use of uncooked cornstarch in children with glycogen storage disorders. Doses of 1–2 gm/kg may be used, but only in children over 9 months old since cornstarch is poorly digested in younger infants. In the absence of controlled studies demonstrating its effectiveness in HI, the Guideline Committee was unable to provide a consensus recommendation on use of cornstarch in children with HI.

## 3. Surgical Management

3.1 We Recommend that Surgery Be Considered for Children Suspected to Have a Resectable Focal Lesion [GRADE 1⊕⊕⊕○]

### 3.2 We Suggest that Infants with Hyperinsulinism due to Diffuse Pancreatic Disease Should Undergo Surgery if Hypoglycemia Is Not Adequately Controlled despite Maximal Medical Therapy [GRADE 2⊕⊕○○]

When genetic tests and radiologic imaging studies suggest the likelihood of a focal lesion, surgical resection is the approach of choice since infants can be cured and avoid either ongoing hypoglycemia or the development of diabetes mellitus. This is especially true if the location of the lesion allows complete resection and if the surgical team has the necessary expertise, including a protocol for intraoperative examination of frozen biopsies to guide the operative strategy [152–154]. For example, in some cases, removal of lesions in the pancreatic head may require a Roux-en-Y pancreatojejunostomy that preserves pancreatic duct connections from the body and tail of the pancreas [87].

At surgery for suspected focal HI, biopsies should be taken from the pancreatic head, body, and tail to clearly establish whether there is diffuse disease versus an underlying focal lesion. Diffuse HI can be recognized by increased numbers of islet cells with large nuclei (nucleomegaly) throughout the pancreas; ascertainment of islet nucelomegaly is ideally undertaken by a pathologist experienced in the recognition of HI. Focal lesions are typically small, 0.5–1 cm in diameter, and contain increased masses of endocrine cells often interspersed with acinar cells and duct structures; biopsies from the remaining pancreas are histologically unremarkable. Focal HI lesions are often unencapsulated and frequently extend into the adjacent normal parenchyma, creating a challenge for assessing involvement of resection margins. In BWSp, there is a similar expansion of islet cell tissue, extending over large areas of the pancreas, making complete resection more difficult. In some cases of HI, histologic changes typical of diffuse HI occur only in localized regions of the pancreas; this has been termed LINE-HI, mosaic, or “atypical” hyperinsulinism and may be associated with somatic variants of HI genes, such as *ABCC8*, *GCK*, and inappropriate expression of *HK1* [80, 85]. Due to the variety of histologic forms of congenital HI, the choice and plan for pancreatic resection should be in close collaboration with a pathologist experienced in HI and with access to a high-quality laboratory and suitable protocols for rapid and accurate review of pancreatic biopsies.

In cases of diffuse HI that have been diagnosed by genetic testing, medical treatment is the first choice; however, if hypoglycemia cannot be adequately controlled, pancreatectomy may be required. For diffuse HI, a 90–98% pancreatectomy is recommended to strike a balance between control of hypoglycemia and the delayed development of diabetes, while preserving bile duct drainage [155, 156].

Following surgery, immunohistochemical staining of permanent sections should be performed using neuroendocrine markers (chromogranin and synaptophysin) to highlight endocrine tissue and for assessment of margin involvement. In focal lesions and in regions of endocrine cell overgrowth in BWSp, loss of maternal heterozygosity at 11p15 can be demonstrated by loss of nuclear p57 staining in lesion cells [157].

The immediate postsurgical management of children with HI may be complicated and, thus, should be in an appropriate intensive care setting. Intraoperative and postoperative plasma glucose fluctuations are common and do not reflect the ultimate glycemic outcome of surgery. However, prevention of postoperative hypoglycemia during this period is important to minimize neuroglycopenia. Plasma glucose levels should be monitored closely, with a goal of preventing hypo or hyperglycemia [158]. Insulin therapy by either subcutaneous injection or intravenous insulin infusion should be started if there is persistent hyperglycemia postoperatively (>250 mg/dL [>14 mmol/L]) [158] that does not respond to fluid therapy or if there is hyperglycemia with hyperketonemia (beta-hydroxybutyrate levels >2 mmol/L). If there is a persisting insulin requirement at the time of conversion to enteral feeding, insulin administration can be transitioned to the subcutaneous route. Conversely, children who cannot be weaned from intravenous glucose-containing infusions without hypoglycemia may require further medical management for persisting hyperinsulinism.

Following pancreatectomy, regardless of underlying diagnosis, all children should be evaluated to determine whether they are euglycemic, have persistent hypoglycemia, or have hyperglycemia requiring insulin treatment. Children who are weaned from IV glucose without hypoglycemia should have a fasting study performed to demonstrate whether they are cured or need further medical management. Details on how to perform either diagnostic or safety/cure fasts are summarized in Tables 4 and 5 [159]. Cure of HI can be demonstrated by the development of hyperketonemia (beta-hydroxybutyrate >1.8 mmol/L) prior to development of hypoglycemia (glucose <50 mg/dL [2.8 mmol/L]). Further resection of pancreatic tissue may be required in medically unmanageable cases [160].

The cure rate of surgery for focal HI is high and is reported to be >95% in some studies [87]. This can be achieved without the risk of subsequent diabetes when just the focal lesion is removed. Following 95–98% pancreatectomy for diffuse disease, hypoglycemia may recur in up to 50–60% of patients: in such cases, additional medical therapies are usually sufficient to achieve euglycemia, although some may require second surgery. Approximately 25% of the patients in the post-operative period have permanent diabetes, and this number can rise to 91% by 14 years of age [86, 87, 161–164].

Children who have undergone greater than 50% pancreatectomy or a pancreatecojejunostomy are at risk for exocrine pancreatic insufficiency requiring pancreatic enzyme replacement [73]. A fecal elastase assay is commonly used for screening [165]. Other recommended monitoring includes plasma levels of fat-soluble vitamins (vitamins A, D, and E) and coagulation tests for vitamin K deficiency [166].

## 4. Discharge Planning

### 4.1 We Suggest that Discharge Planning for Children with Hyperinsulinism Include an Assessment of Fasting Tolerance [GRADE 2⊕⊕○○]

Prior to discharge from the hospital, a fasting study to determine the control of HI is suggested for all children, regardless of whether they are on medical therapy or have undergone pancreatic resection. The fasting duration should be predetermined for each patient to ensure that they will be safe in their home environment and to guide the child’s sleeping, feeding, and glucose monitoring regimen. For those with ongoing hypoglycemia, we suggest gastrostomy tube placement prior to discharge home for use in emergency situations or for continuous overnight feeds/dextrose when fasting tolerance is too short for safe overnight glucose control. Contingency plans should be considered for accidental disconnection of overnight continuous intragastric infusions; these may include the use of a continuous glucose monitoring system and nocturnal enuresis alarm pad (to identify fluid leaks). Individuals with ongoing hypoglycemia should also have access to glucagon therapy for emergency rescue.

An individualized approach should be taken for decision-making regarding child and family readiness for safe discharge home. Upon discharge, any required medications, supplies for glucose monitoring, discharge summary letter/emergency management plans for hypoglycemia/hyperglycemia, and follow-up plans should be provided to the family both in their language and the primary language of the country in which they reside. Other medical and psychological co-morbidities should also be addressed before discharge. In a recent study, caregivers have reported that the ongoing worry associated with managing the complexity of their child’s HI can impact their physical and mental health. Therefore, we suggest providing psychological resources to families and offering them connections to hyperinsulinism patient organizations for support [167]. Referral for genetic consultation and counseling may also be indicated [168].

## 5. Long-Term Management of Patients with Hyperinsulinism

### 5.1 We Suggest Regular Follow-Up and Monitoring to Assess Glycemic Control, Medication Side Effects, and the Development of Diabetes or Pancreatic Insufficiency [Grade 2⊕⊕⊕○]

All children with HI need regular monitoring of their plasma glucose levels at home to guide treatment adjustments. In a patient driven report, more than 65.2% of patients required adjustments to the regimen in the first 3 months following discharge and despite careful follow-up, intermittent hypoglycemia is common [116]. The side effects of the HI medications [111, 128, 169] should be monitored, and support should be provided from the multidisciplinary feeding team in the hospital as well as in the community. In some children with certain forms of HI (e.g., defects in *ABCC8*, *KCNJ11*, *HNF-1A*, and *HNF-4A*), severity may decrease over time [170–172], allowing dose adjustments to medications.

For patients who have undergone a pancreatectomy and are no longer on treatment for hypoglycemia, screening for diabetes should include hemoglobin A1c every 6–12 months and monitoring for symptoms of hyperglycemia. Transition to diabetes may be slow, and some patients may have both fasting hypoglycemia and postprandial hyperglycemia. The need for diabetes medications should be evaluated on an individual basis, taking into account the current HbA1c, ability to fast without hypoglycemia, and presence or absence of symptoms of diabetes.

In patients with postsurgical diabetes mellitus, insulin treatment by conventional or pump therapy similar to children with type 1 diabetes mellitus is necessary to achieve optimal glycemic control [173]. Pancreatic enzyme replacement therapy should be initiated when there is evidence of pancreatic insufficiency [166].

### 5.2 We Recommend that, due to the Increased Risk of Neurocognitive Deficits and Feeding Difficulties, All Children with Hyperinsulinism Should Undergo a Referral for Developmental Surveillance, Feeding Assessment, and Early Intervention [GRADE 1⊕⊕⊕○]

Neurodevelopment delays and neurological disorders, including epilepsy and microcephaly, due to hypoglycemic brain injury occur frequently in patients with HI [163, 164, 172]. Infantile spasms [174]; an increase in motor and speech delay during early childhood [175]; or deficits in attention, memory, visual, and sensorimotor functions have also been reported. Children with transient HI are also at risk of developing neurodevelopmental deficits with reported incidence rates ranging between 26 and 44% [170, 176, 177, 178]. Abnormal neurodevelopment and seizures are particularly high in HI associated with *GLUD1* pathogenic variants [25].

Children with HI should have regular neurodevelopmental follow-up and monitoring that should include formal neurodevelopmental testing during early childhood for appropriate educational and rehabilitative placement. The care of children with HI may need to involve neurodevelopmental pediatricians, physiotherapists, occupational therapists, and speech and language therapists [26].

Feeding problems that may be associated with HI are complex, multifactorial, and often iatrogenic [151]. Feeding issues have been reported in 68.6% of all patients [116]. Vomiting, sucking and swallowing difficulties, and food aversion can occur either in isolation or combination. The use of medications that cause nausea and anorexia and have unpleasant taste, and the use of intravenous glucose support and tube feedings with high carbohydrate content that impair appetite, can interrupt the development of normal feeding milestones and put infants with HI at risk of feeding problems. Although the goal of preventing hypoglycemia is the primary goal in the newborn period, we suggest encouraging oral feeding over tube feeding especially when intravenous glucose support is being used to control hypoglycemia [28]. Prompt management of medical problems, such as vomiting and gastroesophageal reflux, may help prevent long-term feeding problems. In addition, in children with ongoing hypoglycemia, insertion of a gastrostomy tube should be considered for prompt correction of hypoglycemia, and parents should be advised of the possibility of tube dependency and how to avoid this complication.

### 5.3 We Suggest that Programs Be Established for the Transition to Adult Care for Children and Adolescents with Hyperinsulinism and Ongoing Medical Needs, and for the Development of Adult Programs for Adults with Hyperinsulinism [Grade 2⊕⊕○○]

Adults with HI who continue to have complex needs require transition of care to appropriately trained adult specialists to address all ongoing issues, including HI treatment, neurodevelopmental delays and memory issues, hypoglycemia unawareness, risk of developing diabetes, and implications of genetic diagnosis.

## Conclusions

Scientific and clinical advances over the last three decades have improved our understanding of the pathophysiology of HI in children and have led to treatment approaches that take into account the many differences in their genotype and phenotype. Despite these advances, treatment options are still limited for most children with HI. Affected children continue to experience high rates of neurological sequelae due to hypoglycemia-induced brain damage. To improve neurological outcomes, prompt recognition of hypoglycemia and its etiology leading to rapid and effective treatment are essential.

Currently, pancreatectomy may be required for the surgical treatment of diffuse HI. New medical therapies are urgently needed to prevent the need for pancreatectomy and the resultant hypoglycemia and diabetes in the postoperative period. In addition, alternative medical therapy choices are needed to replace those current medications that may cause significant side effects.

Centers caring for infants and children with HI should consider developing multidisciplinary teams to provide all aspects of care, including social and psychological support not only for the children but also the families. An individualized approach to each child would be preferred to ensure optimal patient experience and outcomes. It is also important to offer long-term peer support to patients and families. There are a host of national and international family support organizations that can be found online (https://congenitalhi.org/links/).

These guidelines provide a systematic approach to the diagnosis and management of children with persistent hypoglycemia due to hyperinsulinism while at the same time recognizing that access to diagnostic tools, medications, and medical and surgical expertise is limited in many areas of the world and that good-quality evidence is often lacking. This further illustrates the urgent need for collaborative research efforts to generate this evidence and revise our recommendations in the future.

## Figures and Tables

**Table 1.** Diagnostic features of HI at the time of hypoglycemia (plasma glucose <50 mg/dL [2.8 mmol/L])

Evidence of excessive insulin action at the time of hypoglycemia Suppressed plasma β-hydroxybutyrate (<1.8 mmol/L) Suppressed plasma free fatty acids (<1.7 mmol/L) Inappropriately large glycemic response to glucagon (≥30 mg/dL [≥1.7 mmol/L]) Increased glucose infusion rate required to maintain euglycemia above normal for age >8 mg/kg/min for neonates >3 mg/kg/min for adults Evidence of excessive insulin secretion/inadequate suppression of insulin secretion at the time of hypoglycemia (these are less definitive than evidence of excessive insulin action) Plasma insulin >1.25 μU/mL (8.7 pmol/L) C-peptide >0.5 ng/mL (>0.17 nmol/L)

**Table 2.** Classification of hyperinsulinism disorders in infants and children

	Risk factors	Clinical features
(a) Acquired neonatal HI	Maternal diabetes, including gestational diabetes	Large for gestational age (LGA)
	Perinatal stress-induced HI	Small for gestational age (SGA) Maternal hypertension, pre-eclampsia, eclampsia
	Maternal drugs	Ritodrine, sulfonylurea, high GIR during labor, etc.
(b) Acquired non-neonatal HI	Paraneoplastic HI	Insulinoma *(sporadic or MEN1)*
	Surgically-induced HI	Post-gastric bypass, post-fundoplication for gastroesophageal reflux *(NIPHS: non insulinoma* pancreatogenous *hypoglycemia syndrome)*
	Drug-induced HI	Antidiabetic medications *(Insulin, sulfonylureas)*
	Autoimmune HI (anti-insulin or insulin receptor-activating antibodies)	Spontaneous or associated with drugs or viral infections) Hirata’s disease *(insulin autoimmune syndrome: anti-insulin antibodies post sulphydryl medications: methimazole, carbimazole, alpha-lipoic acid and post measles virus, mumps virus, rubella virus, varicella zoster virus*, coxsackie *B virus and hepatitis C virus)*
	Histology	Genes
(c) Genetic HI: isolated HI	Diffuse form	*ABCC8, KCNJ11, GLUD1, GCK, HNF4A, HNF1A, HADH, SLC16A1, INSR*
	Focal form	Paternally inherited AR variants of *ABCC8* or *KCNJ11*
	LINE- HI (mosaic HI, atypical HI)	Sporadic mosaic AD variants of *ABCC8, GCK, and inappropriate expression of HK1*
	Syndrome	Gene
(d) Genetic HI: syndromic HI	Beckwith-Wiedemann syndrome	Genetic or epigenetic changes of imprinted region 11p15.5. (especially paternal UPD11p; also mutations of imprinting control genes). Pat UPD11p combined with paternal recessive *ABCC8* or *KCNJ11* mutation
	Kabuki syndrome	*KMT2D, KDM6A (usually mosaic)*
	Turner syndrome	Mosaic partial or complete X chromosome monosomy
	See complete list of syndromal HI in table 2	
(e) HI mimickers: hypoinsulinemic hypoketotic hypoglycemia	Autoimmune mimicker	Insulin resistance syndrome type B *(anti-insulin receptor antibodies post viral infection (HIV, HTLV1, hepatitis C) or lymphoproliferative disease, or autoimmune disease (lupus))*
	Paraneoplastic secretion of pro-IGF2	Non-islets cells tumor hypoglycemia (NICTH, Doege-Potter syndrome)
	Genetic disorders of insulin signaling	Mutations in *AKT2, AKT3, PIK3CA, PIK3R2, CCND2, INSR*.
	Fatty acid oxidation disorders	Abnormalities in the carnitine cycle, beta-oxidation, electron transfer, and ketone synthesis

**Table 3.** The genetic etiology of hyperinsulinism

Gene/genetic loci	Phenotype MIM number	Phenotype		Mode of inheritance	Histology	Ref
*ABCC8*	# 256450	Isolated		AR	Diffuse	[25]
				AD/mosaic	Diffuse	[26]
					Atypical	[27, 28]
				AR LOH*	Focal	[29]
	# 606528	Hyperinsulinism with enteropathy and deafness		AR (contiguous deletion including *USH1C)*	Diffuse	[30]
*ADK*	# 614300	ADK deficiency syndrome		AR	Diffuse	[31]
*ALG3*	# 601110	Congenital disorder of glycosylation type 1d		AR	Diffuse	[32]
*ALG6*	# 603147	Congenital disorder of glycosylation type 1c		AR	Diffuse	[33]
*CACNA1C*	# 601005	Timothy syndrome		AD	Diffuse	[34]
*CACNA1D*	# 615474	PASNA syndrome		AD	Diffuse	[35]
*CDKN1C*	# 130650	Beckwith-Wiedemann Spectrum		AD	Diffuse	[36]
*CREBBP*	# 180849	Rubinstein Taybi syndrome 1		AD	Diffuse	[37]
*DIS3L2*	# 267000	Perlman syndrome		AR	Diffuse	[38]
*EIF2S3*	# 300148	MEHMO syndrome		XLR	Diffuse	[39]
*EP300*	# 613684	Rubinstein Taybi syndrome 2		AD	Diffuse	[37]
*FAH*	# 276700	Tyrosinaemia type I		AR	Diffuse	[40]
*FOXA2*	-	Syndromic		AD	Diffuse	[41]
*GCK*	# 602485	Isolated		AD/mosaic	Diffuse/atypical	[42]
*GLUD1*	# 606762	Hyperinsulinism-hyperammonaemia syndrome		AD	Diffuse	[43]
*GPC3*	# 312870	Simpson-Golabi-Behmel syndrome		XLR	Diffuse	[44]
*HADH*	# 609975	Isolated		AR	Diffuse	[45]
*HK1*	-	Isolated		AD	Diffuse	[46]
*HNF1A*	# 600496	Isolated		AD	Diffuse	[21]
*HNF4A*	# 125850	Isolated		AD	Diffuse	[19]
	# 616026	Fanconi renotubular syndrome		AD (p.Arg76Trp)	Diffuse	[21]
*HRAS*	# 218040	Costello syndrome		AD	Diffuse	[47]
*INSR*	# 609968	Isolated		AD	Diffuse	[48]
*KCNJ11*	# 601820	Isolated		AR	Diffuse	[49]
				AD	Diffuse	[50]
				AR LOH*	Focal	[29]
*KCNQ1*	# 192500	Long QT syndrome		AD	Diffuse	[51]
*KMT2D*	# 147920	Kabuki syndrome		AD	Diffuse	[52]
*KDM6A*	# 300867	Kabuki syndrome		XLD	Diffuse	[53]
*MPI*	# 602579	Congenital disorder of glycosylation type 1b		AR	Diffuse	[54]
*PGM1*	# 614921	Congenital disorder of glycosylation type 1 t		AR	Diffuse	[55]
*PMM2*	# 212065	Congenital disorder of glycosylation type 1a	AR		Diffuse	[56]
	-	HI and polycystic kidney disease	AR (c.−167G>T)		Diffuse	[57]
*PHOX2B*	# 209880	Central hypoventilation syndrome	AD		Diffuse	[58]
*NSD1*	# 117550	Sotos syndrome	AD		Diffuse	[59]
*SLC16A1*	# 610021	Isolated (exercise-induced HI)	AD		Diffuse	[60]
*TRMT10A*	# 616033	Syndromic	AR		Diffuse	[61]
*YARS1*	# 619418	Yars-related disease	AR		Diffuse	[62]
Chr5q35 deletion	# 117550	Sotos syndrome	AD		Diffuse	[63]
Chr9p24 deletion	# 151870	9p deletion syndrome	AD		Diffuse	[64]
Chr11p15 imprinting abnormality	# 130650	Beckwith-Wiedemann Spectrum	Sporadic		Diffuse	[65]
Chr13 trisomy	-	Patau syndrome	Sporadic		Diffuse	[66]
ChrX monosomy	-	Turner syndrome	Sporadic		Diffuse	[67]

* Loss of the maternal 11p15 allele within pancreas in combination with a paternally inherited recessive *ABCC8* or *KCNJ11* mutation.

**Table 4.** Diagnostic fasting test

Perform test only on a unit with trained medical/nursing staff who are experienced in the performance of fasting studies Have IV access and D10% (2–5 mL/kg) for emergency resuscitation Measure glucose by POC meter every 2–3 h until glucose <70 mg/dL (<3.9 mmol/L); then every 2 h until <60 mg/dL (<3.3 mmol/L); then hourly until ≤50 mg/dL (<2.8 mmol/L) When glucose <60 mg/dL (<3.3 mmol/L) send specimen for laboratory confirmation of plasma glucose Measure beta-hydroxybutyrate every 2–3 h and when glucose <50 mg/dL (<2.8 mmol/L) When plasma glucose ≤50 mg/dL (<2.8 mmol/L) draw blood for the CRITICAL sample Glucose, insulin, beta-hydroxybutyrate, free fatty acids Ammonia, cortisol, growth hormone, lactate, acyl carnitine profile, urine organic acids Special circumstances: C-peptide, proinsulin, sulphonylurea screen, toxicology screen, serum amino-acids Perform Glucagon Stimulation Test once CRITICAL samples are obtained Measure glucose using POC meter and then give glucagon 30 μg/kg or 0.5–1 mg by IM or IV push as long as glucose is < 50 mg/dL (<2.8 mmol/L) Monitor glucose using POC meter every 10 min for 40 min Terminate test if glucose is still below 50 mg/dL (<2.8 mmol/L) after 30 min After 40 min, may feed and resume treatment to maintain plasma glucose >70 mg/dL (3.9 mmol/L)

**Table 5.** Safety/cure fasting test

Have blood drawing IV line in place Check glucose (POC meter) and beta-hydroxybutyrate every 2–3 h until glucose <70 mg/dL; then every 2 h until <60 mg/dL; then hourly until <50 mg/dL When glucose <60 mg/dL (by POC meter), send specimen for laboratory confirmation of plasma glucose Terminate fast when Plasma BOHB >2 mmol/L on two separate samples 1 h apart Plasma glucose <50 mg/dL Duration of fasting >18 h in <1 year old or >36 h in children 1–10 years old or 72 h in >10 years old
